# Convergent Transcription of Interferon-stimulated Genes by TNF-α and IFN-α Augments Antiviral Activity against HCV and HEV

**DOI:** 10.1038/srep25482

**Published:** 2016-05-06

**Authors:** Wenshi Wang, Lei Xu, Johannes H. Brandsma, Yijin Wang, Mohamad S. Hakim, Xinying Zhou, Yuebang Yin, Gwenny M. Fuhler, Luc J. W. van der Laan, C. Janneke van der Woude, Dave Sprengers, Herold J. Metselaar, Ron Smits, Raymond A. Poot, Maikel P. Peppelenbosch, Qiuwei Pan

**Affiliations:** 1 Department of Gastroenterology and Hepatology, Postgraduate School Molecular Medicine, Erasmus MC-University Medical Center, Rotterdam, 3015 CE, The Netherlands; 2 Department of Cell Biology, Medical Genetics Cluster, Erasmus MC-University Medical Center, Rotterdam, 3015 CE, The Netherlands; 3 Department of Microbiology, Faculty of Medicine, Gadjah Mada University, Yogyakarta, Indonesia; 4 Department of Surgery, Postgraduate School Molecular Medicine, Erasmus MC-University Medical Center, Rotterdam, 3015 CE, The Netherlands

## Abstract

IFN-α has been used for decades to treat chronic hepatitis B and C, and as an off-label treatment for some cases of hepatitis E virus (HEV) infection. TNF-α is another important cytokine involved in inflammatory disease, which can interact with interferon signaling. Because interferon-stimulated genes (ISGs) are the ultimate antiviral effectors of the interferon signaling, this study aimed to understand the regulation of ISG transcription and the antiviral activity by IFN-α and TNF-α. In this study, treatment of TNF-α inhibited replication of HCV by 71 ± 2.4% and HEV by 41 ± 4.9%. Interestingly, TNF-α induced the expression of a panel of antiviral ISGs (2-11 fold). Blocking the TNF-α signaling by Humira abrogated ISG induction and its antiviral activity. Chip-seq data analysis and mutagenesis assay further revealed that the NF-κB protein complex, a key downstream element of TNF-α signaling, directly binds to the ISRE motif in the ISG promoters and thereby drives their transcription. This process is independent of interferons and JAK-STAT cascade. Importantly, when combined with IFN-α, TNF-α works cooperatively on ISG induction, explaining their additive antiviral effects. Thus, our study reveals a novel mechanism of convergent transcription of ISGs by TNF-α and IFN-α, which augments their antiviral activity against HCV and HEV.

Cytokines orchestrate cellular communication in an autocrine, juxtacrine, or paracrine fashion through binding to distinct families of receptors, triggering specific immune responses against invading pathogens. The interferon (IFN)-mediated innate immune response is probably the most prominent response and provides a robust first defense line. Among different types of interferons, IFN-α (a type I member) has been used for decades to treat chronic hepatitis B or C infection in the clinic^1^. When stimulated by its cognate ligand, interferon receptors respond by the activation of kinases of the Janus family (JAKs), which in turn phosphorylate tyrosine residues in the intracellular tail of the interferon receptors. These phosphotyrosines serve as docking sites for recruitment and phosphorylation of the Signal Transducers and Activators of Transcription (STAT) family, which provokes STAT1 and STAT2 dimerization and subsequent binding to interferon regulatory factor 9 (IRF9) to form the IFN-stimulated gene factor 3 (ISGF3) complex. The ISGF3 complex translocates into the nucleus, and binds to specific promotor elements denoted as interferon signaling response elements (ISREs) and thus mediate the transcription of so-called interferon-stimulated genes (ISGs). ISGs are the ultimate antiviral effectors of the interferon signaling.

It is generally believed that ISGs are predominantly induced by interferons. However, ISGs are still up-regulated in embryonic fibroblasts from IFN alpha/beta receptor knockout mouse upon infection of West Nile virus^2^. These observations suggest the existence of alternative mechanisms of regulating ISG transcription. But these non-canonical mechanisms remain largely unknown.

Tumor necrosis factor alpha (TNF-α is another important cytokine that mediates host response to infections. TNF-α /TNFR interactions can play decisive roles in the outcome of a number of viral infections, contributing to virus control or immune mediated pathology^3^. Deregulation of TNF-α is associated with many pathological conditions, including various types of arthritis and inflammatory bowel disease (IBD)^4^. TNF-α inhibitors have been successfully used in the clinic to treat these chronic immune-mediated diseases^5^. However, patients receiving TNF-α inhibitors are often at high risk of viral infections^6^. Treatment with TNF-α inhibitors have been reported to increase reactivation of concurrent chronic hepatitis B and potentially increase hepatitis C virus (HCV) replication^7^, further supporting the importance of TNF-α in defending the human body against viral infections. Interestingly, several previous studies reported crosstalk between TNF-α and the antiviral interferon signaling and ISG expression in the setting of vesicular stomatitis virus^8^, hepatitis C virus (HCV)^9^, respiratory virus^10^ and poxvirus infections^11^.

However, the exact antiviral mechanisms of TNF-α and how it cooperates with the interferon signaling remain largely elusive, thus prompting us to explore their molecular basis. Here we report that TNF-α alone was sufficient to induce the expression of ISGs and to exert antiviral activity against HCV and hepatitis E virus (HEV). This is through the activation of the NF-κ B signaling but independent of the canonical interferon pathway. Surprisingly, we found a consensus DNA binding sequence between the NF-κ B and ISRE motif with bioinformatics analysis. Functional assays revealed that the NF-κ B complex is able to bind to the ISRE motif and directly activates the transcription of antiviral ISGs. Combination of TNF-α with IFN-α further boosts the induction of ISGs and results in augmented antiviral activity against HCV and HEV. Thus, this study identified a non-canonical mechanism of driving antiviral ISG transcription, which provides the molecular basis for the antiviral action of TNF-α and its additive antiviral effect with interferon.

## Results

### TNF-α activates ISG transcription and exerts antiviral activity against HCV and HEV

**Figure 1:**
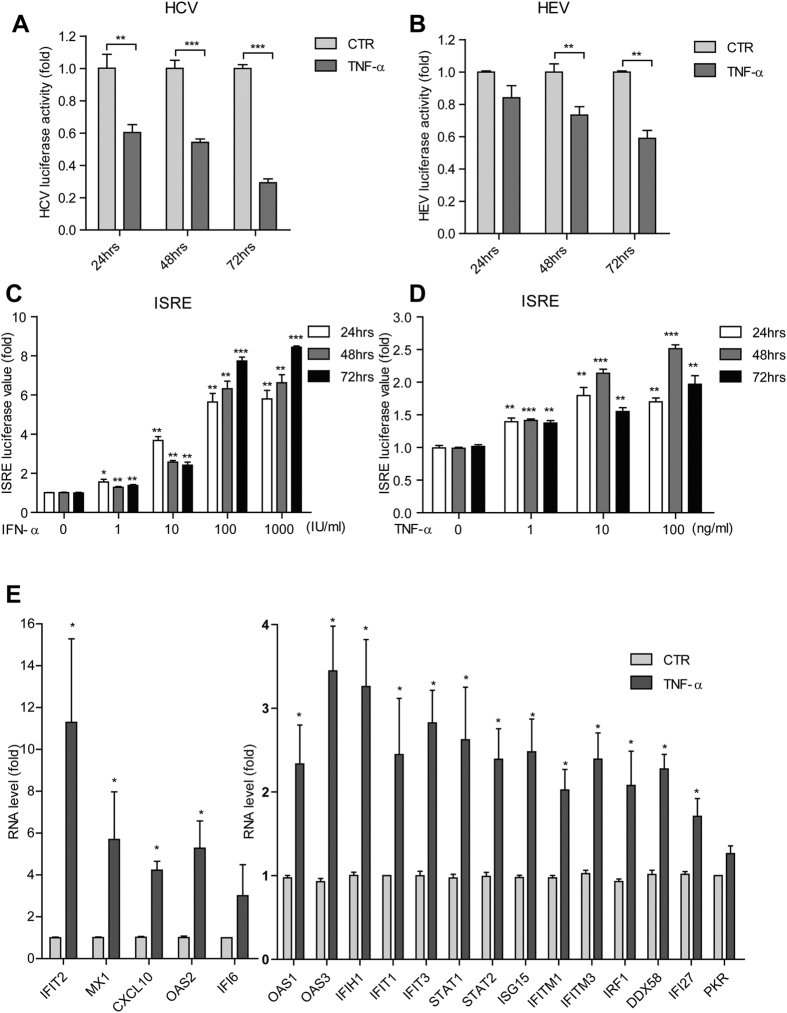
TNF-α activates ISG transcription and exerts antiviral activity against HCV and HEV. (**A**) In the Huh7 cell-based subgenomic HCV replicon, treatment with recombinant human TNF-α (100 ng/ml) inhibited HCV replication-related luciferase activity as measured at 3 different time points (n =  5). (**B**) Same as (**A**) for the Huh7 cell-based subgenomic HEV replicon model. (**C**) In the Huh7 cell-based ISRE luciferase reporter cells, treatment with IFN-α resulted in a dose-dependent induction of ISRE-related luciferase activity (n =  3 independent experiments with 2–3 replicates each). (**D**) Same as (**C**) for TNF-α . (**E**) Expression profile of 20 antiviral ISGs in Huh7 cells as measured by qRT-PCR. Most ISGs were highly up-regulated with TNF-α treatment (n =  5). Data presented as mean ±  SD (*P <  0.05; **P <  0.01; ***P <  *0.001*).

TNF-α is involved in host responses to a variety of pathogen invasions, including HCV and HEV infections^9^,^12^. To assess the direct effects of TNF-α on HCV and HEV replication, we employed a human hepatocyte cell line, i.e. Huh7, transfected with a HCV or HEV replicon luciferase as reporters. In parallel, Huh7 cells constitutively expressing a non-secreted firefly luciferase under control of the human phosphoglycerate kinase (PGK) promoter (LV-PGK-Luc) were also used for normalization of nonspecific effects on luciferase signals. Both HCV and HEV replicon luciferase activity were significantly inhibited by treatment of cells with TNF-α (Fig. 1A,B). For instance, 100 ng/ml TNF-α inhibited HCV to 29 ±  2.4% (n =  5, *P* <  0.001), HEV to 59 ±  4.9% (n =  5, *P* <  0.01) at 72 hrs.

Since TNF-α has been reported to interacts with interferon signaling and ISGs are the ultimate antiviral effectors of the interferon cascade, we thus attempted to investigate whether TNF-α alone has any effect on ISG transcription. Based on the knowledge that interferon induces ISG expression via the activation of the ISRE motifs within the promoters of ISGs, a Huh7 cell line stably harboring a ISRE-driven luciferase reporter was used^13^. As expected, IFN-α treatment induced a strong transactivation of ISRE-driven luciferase value (Fig. 1C). Surprisingly, TNF-α stimulation also provoked a strong transactivation of the ISRE transcription elements (Fig. 1D). This interesting result prompted us to investigate the relative expression level of a panel of well-studied antiviral ISGs by qRT-PCR. Consistently, treatment of TNF-α provoked the induction of most tested ISGs, ranging from 1.7 to 11.3 fold increase (Fig. 1E). These data demonstrate that TNF-α transactivates the ISRE motif, resulting in the induction of ISGs, which in turn mediate the antiviral effects of TNF-α against HCV and HEV.

### Activation of ISRE transcription by TNF-α does not require interferon production

**Figure 2:**
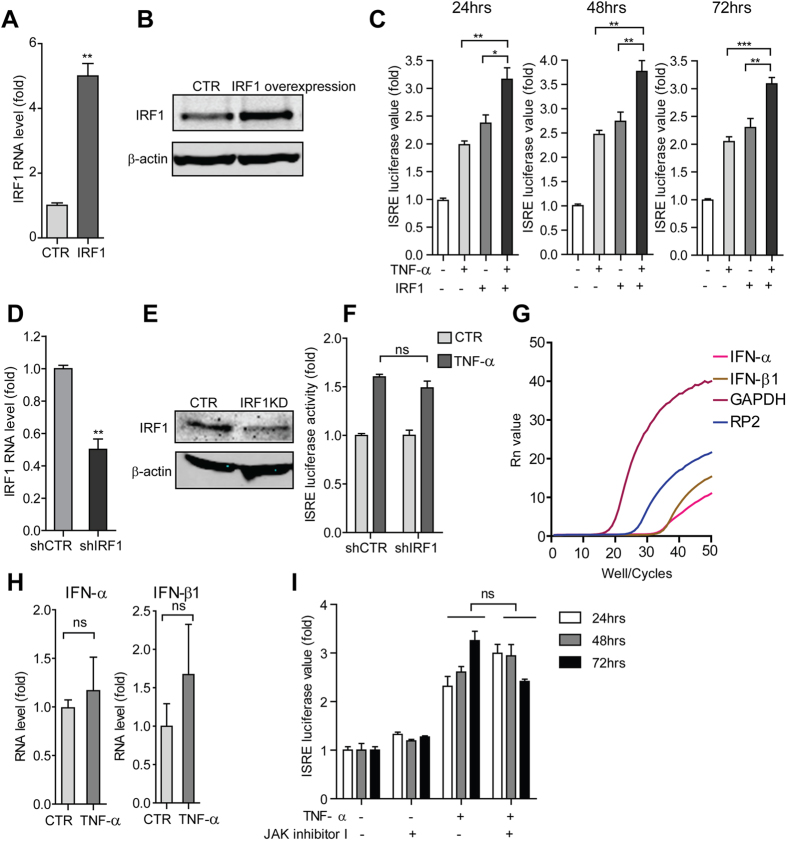
Activation of ISRE transcription by TNF-α does not require interferon production and the JAK-STAT signaling. (**A**) qRT-PCR analysis of IRF1 overexpression by lentiviral vectors in the Huh7 based ISRE luciferase reporter cells. Compared to the control vector transduced cells, the IRF1 lentiviral vector showed strong IRF1 induction on RNA level. (**B**) Western blot analysis confirmed the successful overexpression of IRF1 by lentiviral vectors in the Huh7 based ISRE luciferase reporter cells. (**C**) In the Huh7 cell-based ISRE luciferase reporter cells, the combination of IRF1 over-expression and TNF-α induced a strong additive ISRE activation as measured at 3 different time points (n =  5). (**D**) qRT-PCR analysis of IRF1 knockdown by lentiviral shRNA vectors in the Huh7 based ISRE luciferase reporter cells. Compared to the control vector transduced cells, the IRF1 shRNA treated clones showed strong reduction of IRF1 RNA levels. (**E**) Western blot analysis confirmed the successful knockdown of IRF1 by lentiviral shRNA vectors in the Huh7 based ISRE luciferase reporter cells. (**F**) Knockdown of IRF1 in Huh7 based ISRE luciferase reporter cells did not block TNF-α induced ISRE-related luciferase activation (n =  4). (**G**) The relative IFN-α and β 1 expression levels in Huh7 cells were determined by qRT-PCR. GAPDH and RP2 served as internal reference genes. (**H**) IFN-α and β 1 expression levels in Huh7 cells were not up-regulated upon TNF-α treatment as measured by qRT-PCR (n =  6). (**I)** JAK inhibitor I (5 μ m) did not abrogate TNF-α induced ISRE-related luciferase activation (n =  3 independent experiments with 2–3 replicates each). Data presented as mean ±  SD (*P <  0.05; **P <  0.01; ***P <  *0.001;* ns, not significant).

The fact that TNF-α can induce ISGs inspired us to investigate the straightforward possibility that TNF-α merely triggers the production of interferons. Interferon regulatory factor 1 (IRF1) was demonstrated to be important in a TNF-α triggered IFN-β autocrine loop in primary macrophage cells^14^. To dissect whether a similar mechanisms exist in our experiment system, we first studied the potential involvement of IRF1. Lentiviral vector was used to over-express IRF1 in Huh7 based ISRE-driven luciferase reporter cells and the successful over-expression of IRF1 was confirmed at both mRNA and protein levels (Fig. 2A,B). IRF1 over-expression significantly increased ISRE-regulated luciferase activity (Fig. 2C). Surprisingly, the combination of IRF1 over-expression and TNF-α induced a strong additive ISRE activation (Fig. 2C). Furthermore, stable IRF1 knockdown by lentiviral RNAi (Fig. 2D,E) had no significant effect on TNF-α induced ISRE activation (Fig. 2F). In addition, the involvement of another interferon regulatory factor, IRF7, was also examined via loss-of-function assay. TNF-α induced ISRE activation was not affected even upon the efficient IRF7 knockdown (Supplementary Figure 1A–C). These results suggest that TNF-α triggered ISRE activation is independent of IRF1 and IRF7.

We next investigated the effects of TNF-α on gene expression of type I interferons. As determined by qRT-PCR, the constitutive expression levels of IFN-α and β 1 in Huh7 cells are rather low, compared to the reference genes GAPDH and RP2 (Fig. 2G). Moreover, TNF-α treatment did not significantly increase IFN-α and IFN-β 1 mRNA levels (Fig. 2H). This is consistent with a previous study showing that the Huh7 cell line responds to interferon but does not produce interferon^15^. These data collectively indicate that activation of ISRE transcription by TNF-α does not require interferon production in our model system.

### TNF-α induced ISRE activationh is independent of the JAK-STAT signaling

Classically, ISGs are induced by interferons via the JAK-STAT signaling. Following receptor activation by interferons, JAK1 phosphorylates STAT1 and Tyrosine kinase 2 (TYK2) phosphorylates STAT2. This provokes STAT1 and STAT2 dimerization and subsequent binding to IRF9 to form the ISGF3 complex. The ISGF3 complex translocates into the nucleus, binds to the ISRE motif [5′ -CAGTTTCACTTTCC-3′ ] and drives the transcription of ISGs (Supplementary Figure 2A). To test whether activation of ISRE by TNF-α require JAK-STAT signaling, we first examined the role of JAKs. Strikingly, neither JAK inhibitor I (an inhibitor of JAK1, JAK2, JAK3 and TYK2) nor Bayer-18 (a selective TYK2 inhibitor) abrogated TNF-α induced ISRE activation (Fig. 2I and Supplementary Figure 2B). Consistently, TNF-α induced ISG expression was not affected by the treatment of JAK inhibitor I (Supplementary Figure 2D). In contrast, both IFN-α induce ISRE activation and ISG expression were largely blocked by JAK inhibitor I (Supplementary Figures 2C and 3A). Interestingly, the selective TYK2 inhibitor, Bayer-18, did not significantly affect IFN-α induced ISRE activation (Supplementary Figure 2C). This is consistent with a previous study, showing that TYK2 plays a restricted role in IFN-α signaling^16^.

**Figure 3:**
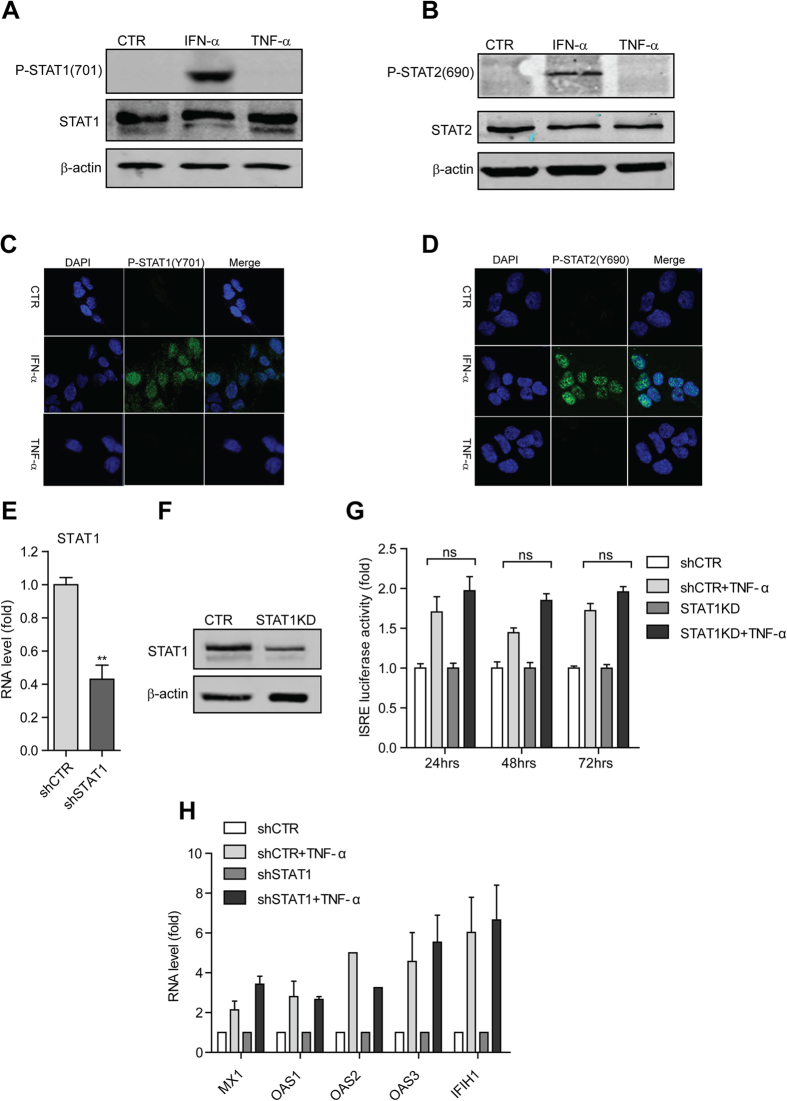
TNF-α activates ISRE in a STAT1 independent manner. (**A**) Western blot analysis of total STAT1 and phosphorylated STAT1 (Y701P) protein levels under the treatment of TNF-α (100 ng/ml), IFN-α (1000IU). (**B**) Same as (**A**) for the detection of total STAT2 and phosphorylated STAT2 (Y690P) protein levels under the treatment of TNF-α , IFN-α . (**C**) Confocal microscopy analysis of phosphorylated STAT1 (Y701P) localization in Huh7 cells treated with IFN-α or TNF-α . STAT1 was phosphorylated and translocated to the nucleus upon IFN-α , but not TNF-α treatment. Phosphorylated STAT1 (Y701P) antibody (green). Nuclei were visualized by DAPI (blue). (**D**) Same as (**C**) for the detection and localization of phosphorylated STAT2 (Y690P). (**E**) qRT-PCR confirmed the successful STAT1 knockdown by lentiviral shRNA vectors in the Huh7 based ISRE luciferase reporter cells. (**F**) Western blot analysis confirmed the successful knockdown of STAT1 by lentiviral shRNA vectors in the Huh7 based ISRE luciferase reporter cells. (**G**) STAT1 knockdown had no significant influence on TNF-α induced ISRE-related luciferase activation as measured at 3 different time points (n =  3 independent experiments with 2–3 replicates each). (**H**) STAT1 knockdown exerts no effect on TNF-α induced ISG expression as measured by qRT-PCR (n =  3).

Furthermore, to see if TNF-α treatment has any effect on STATs activation and translocation, we examined the phosphorylation status of STAT1 at amino acid 701 (Y701P) and STAT2 at amino acid 690 (Y690), which are indispensable signature of STAT1 and STAT2 activation, respectively. WB results showed TNF-α treatment had no effects on the phosphorylation of both STAT1 and STAT2 at indicated sites (Fig. 3A,B). Confocal microscopy analysis also confirmed that IFN-α induced the activation and nuclear translocation of STAT1 and STAT2 via the phosphorylation at indicated sites, while TNF-α had no effects (Fig. 3C,D). To further exclude a role of STAT1 in TNF-α induced ISRE activation, lentiviral RNAi was used to knockdown STAT1. The stable STAT1 knockdown (Fig. 3E,F) had no effect on both TNF-α induced ISRE activation and ISG expression (Fig. 3G,H). Collectively, TNF-α triggered ISRE activation is totally independent of STAT1.

In addition, the role of IRF9 was also verified, which is a key downstream element of interferon pathway. IRF9 was up-regulated and translocated into cell nucleus upon IFN-α stimulation, whereas TNF-α stimulation did not induce the translocation of IRF9 into cell nucleus (Supplementary Figure 3B). These results collectively demonstrate that TNF-α induced ISRE activation is independent of the JAK-STAT signaling.

### TNF-α activates ISRE via TNF receptor 1

**Figure 4:**
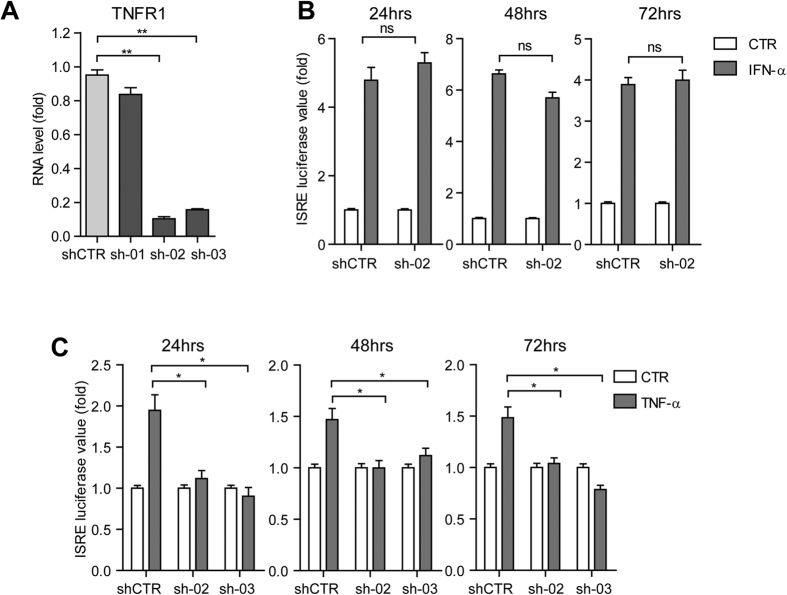
TNF-α activates ISRE via TNF receptor I. (**A**) qRT-PCR analysis of TNFR1 knockdown by lentiviral shRNA vectors in the Huh7 based ISRE luciferase reporter cells. Compared to the control vector transduced cells, the two shRNA treated clones (sh-02 and sh-03) showed strong reduction of TNFR1 RNA levels. (**B**) TNFR1 knockdown had no significant influence on IFN-α induced ISRE-related luciferase activation as measured at 3 different time points (n =  3 independent experiments with 2–3 replicates each). (**C**) TNFR1 knockdown blocked TNF-α induced ISRE-related luciferase activation as measured at 3 different time points (n =  3 independent experiments with 2–3 replicates each).

TNF receptor (TNFR) is the important upstream component in TNF-α induced signaling transduction. TNF acts through two receptors, TNFR1 and TNFR2. TNFR1 is the major signaling receptor for TNF-α and is expressed by all human tissues, while TNFR2 is mostly expressed in immune cells and mediates limited biological responses^17^. In light of the fact that TNF-α is capable of activating ISG transcription, we sought to determine whether this action of TNF-α was mediated via TNFR. For this, the ISRE reporter cell line was transduced with integrating lentiviral RNAi vectors to silence TNFR1, resulting in a profound down-regulation of TNFR1 expression (Fig. 4A). As expected, IFN-α induced ISRE activation was not influenced (Fig. 4B), but TNF-α induced ISRE luciferase activity was largely abrogated in TNFR1 knockdown cells when compared to control cells (Fig. 4C). Consistently, the induction of ISGs by TNF-α was also blocked by TNFR1 knockdown (Supplementary Figure 4A).

**Figure 5:**
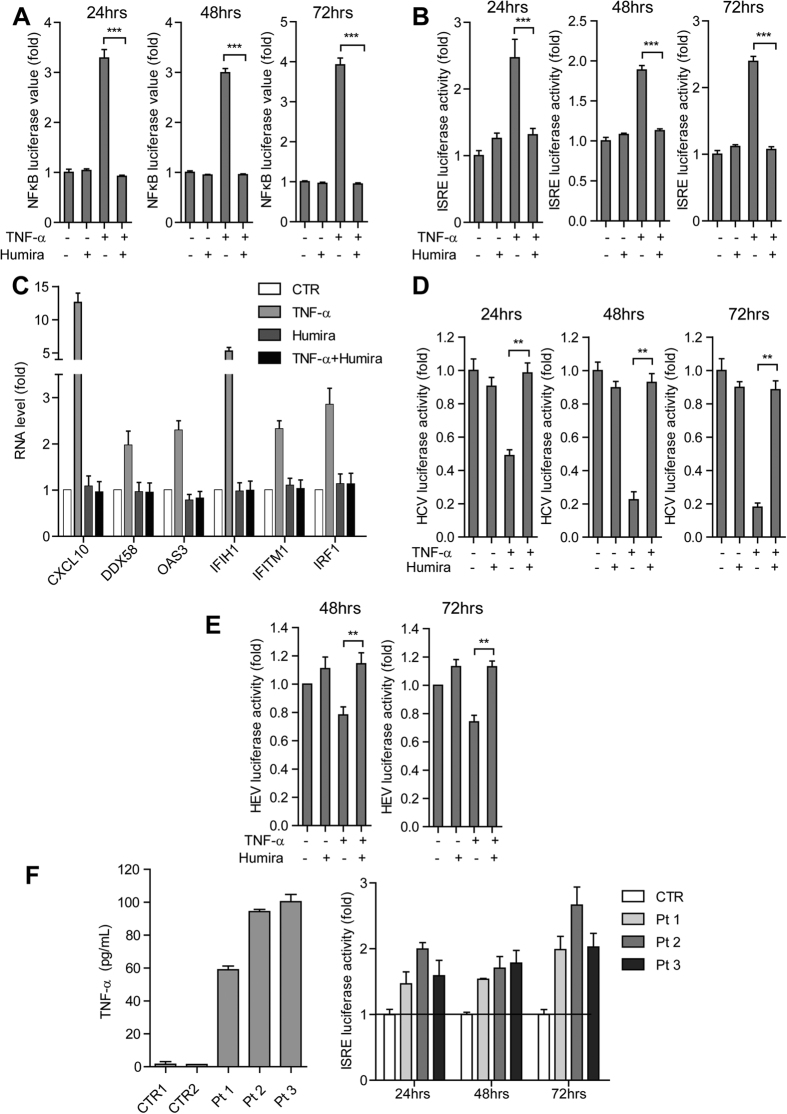
Both TNF-α induced ISG expression and antiviral activity against HCV and HEV were abrogated by its inhibitor Humira. (**A**) In the Huh7 cell-based NF-κ B luciferase reporter cells, the TNF-α inhibitor, Humira, abrogated TNF-α induced NF-κ B-related luciferase activation as measured at 3 different time points (n =  3 independent experiments with 2–3 replicates each). (**B**) Same as (**A**) for the Huh7 cell-based ISRE luciferase reporter cells. (**C**) In Huh7 cells, the TNF-α inhibitor, Humira, abrogated TNF-α induced ISG expression as measured by qRT-PCR (n =  4). (**D**) In the Huh7 cell-based subgenomic HCV replicon, Humira abrogated the TNF-α induced anti-HCV effect as measured at 3 different time points (n =  3 independent experiments with 2–3 replicates each). (**E**) Same as (**D**) for Huh7 cell-based subgenomic HEV replicon. (**F**) TNF-α levels in serum samples collected from anti-TNF-α treatment naive Crohn’s disease patients were measured by ELISA kit (left). Serum samples with higher TNF-α levels showed stronger ISRE-related luciferase activity compared with control serum as measured at 3 different time points. Data presented as mean ±  SD. (*P <  0.05; **P <  0.01; ***P <  *0.001;* ns, not significant).

To further confirm these results, the clinically widely used drug for rheumatoid arthritis patients and Crohn′ s disease, Humira (adalimumab), was used. Humira binds specifically to TNF-α and blocks its interaction with TNF receptors. As expected, Humira effectively blocks TNF-α induced activation of NF-κ B luciferase activity (Fig. 5A), NF-κ B activity being a well-known downstream effect of TNF-α receptor ligation. Importantly, both TNF-α induced ISRE luciferase activity and ISG expression were also abrogated by Humira treatment (Fig. 5B,C). This effect was not limited to Huh7 cells, but also observed in a human lung cell line, A549 (Supplementary Figure 4B). More relevantly, Humira totally abolished TNF-α mediated antiviral effect against HCV and HEV (Fig. 5D,E), providing a possible explanation for the high risk of infection in patients treated with TNF-α inhibitors. Next, we collected serum samples from anti-TNF-α treatment naive Crohn’s disease patients and measured the serum TNF-α levels by ELISA. 3 serum samples with high TNF-α levels were selected to treat Huh7 based ISRE-driven luciferase reporter cells (Fig. 5F). Consistently, all 3 serum samples exerted higher ISRE activity compared to control serum sample (Fig. 5F, right). Furthermore, Humira decreased the serum induced ISRE activity (Supplementary Figure 4C). More interestingly, serum samples with higher TNF-α levels inhibited HCV-related luciferase activity compared to control serum sample (Supplementary Figure 4D). Collectively, these results demonstrate that TNF-α acts via its receptor to activate ISG transcription and exerts antiviral activity, which can be blocked by clinically used TNF-α inhibitor.

### TNF-α mediates the activation of ISRE through NF-κB signaling

Activation of NF-κ B signaling is one of the most important canonical responses to the stimulation of TNF-α . Following TNF receptor activation by TNF-α , inhibitor of kappa B (IkB) proteins undergo phosphorylation dependent ubiquitination and degradation, resulting in the activation and translocation of NF-κ B dimers into the cell nucleus. In the cell nucleus, NF-κ B dimers bind to the specific NF-κ B motifs, [5′ -GGGAA/CTTTCC-3′ ], within the promoter regions driving the expression of NF-κ B target genes (Supplementary Figure 5A). Because some studies have reported that TNF-α can also increase the transcriptional activity of activator protein-1 (AP-1) in some specific cell types^18^,^19^, we thus created Huh7 based stable NF-κ B or AP-1 driven luciferase reporter cell lines, respectively. As shown in Supplementary Figure 5B, stimulation with TNF-α led to strong activation of NF-κ B luciferase activity, but no significant effect on AP-1 activity. Therefore, we only focused on NF-κ B signaling for the following investigation.

**Figure 6:**
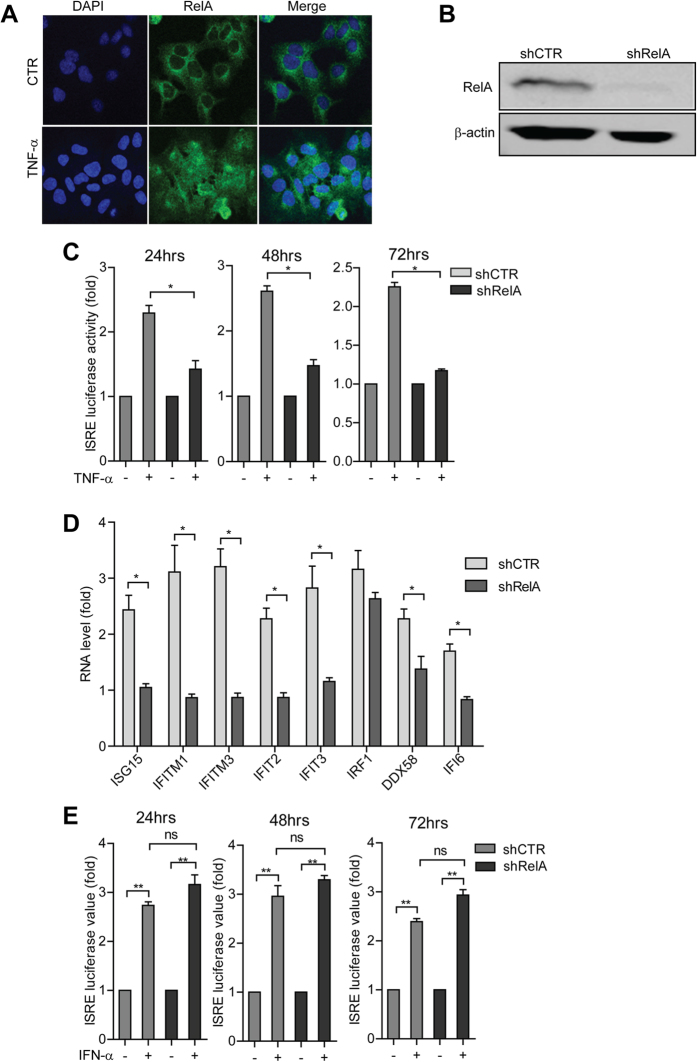
TNF-α mediates the induction of ISRE by activation of the NF-κB signaling. (**A**) Confocal microscopy analysis of RelA induction and localization in Huh7 cells treated with TNF-α . RelA was induced and translocated to the nucleus upon TNF-α treatment. RelA antibody (green). Nuclei were visualized by DAPI (blue). (**B**) Western blot analysis confirmed the successful knockdown of RelA by lentiviral shRNA vectors in the Huh7 based ISRE luciferase reporter cells. (**C**) RelA knockdown largely blocked TNF-α induced ISRE-related luciferase activation as measured at 3 different time points (n =  3 independent experiments with 2–3 replicates each). (**D**) RelA knockdown largely blocked TNF-α induced ISG expression as measured by qRT-PCR (n =  4). (**E**) RelA knockdown has no significant influence on IFN-α induced ISRE-related luciferase activation as measured at 3 different time points (n =  3 independent experiments with 2–3 replicates each). Data presented as mean ±  SD (*P <  0.05; **P <  0.01; ***P <  *0.001;* ns, not significant).

The NF-κ B complex is the endpoint of its signal transduction, which comprises the heterodimeric RelA (P65)-P50 complex. Indeed, unstimulated cells display little nuclear RelA, but the RelA protein level in the cell nucleus was substantially elevated following TNF-α stimulation (Fig. 6A). Thus, to dissect the role of the RelA (P65) - P50 complex in TNF-α induced ISRE activation, the Huh7 ISRE reporter cell line was transduced with integrating lentiviral RNAi vectors to silence RelA (P65), resulting in profound down-regulation of RelA expression (Fig. 6B). Consistently, TNF-α induced ISRE luciferase activity and ISG expression was largely demolished in RelA knockdown cells when compared with control cells (Fig. 6C,D). On the contrary, IFN-α induced ISRE activation was not affected (Fig. 6E). Thus, NF-κ B signaling appears to be essential for TNF-α mediated ISRE activation.

### The NF-κB complex directly binds to ISRE and drives its transcriptional activity

**Figure 7:**
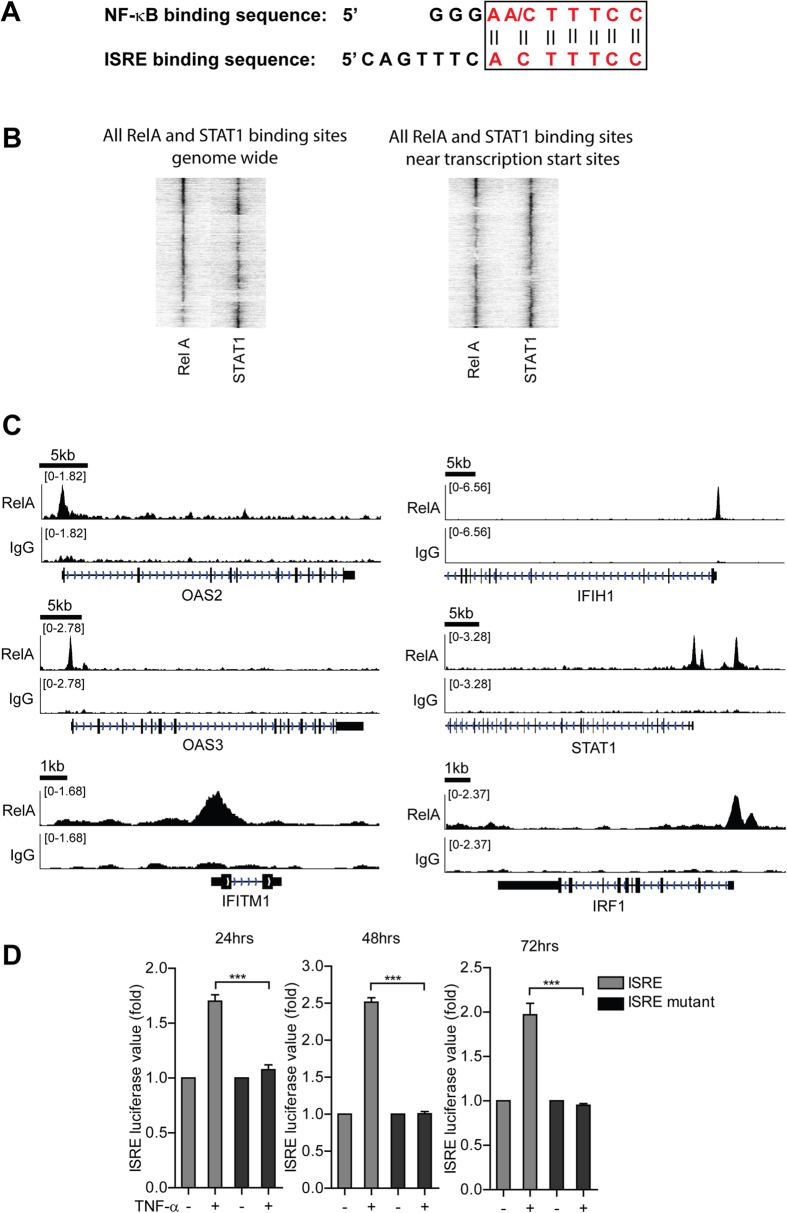
The NF-κB complex directly binds to ISRE and drives its transcriptional activity. (**A**) NF-κ B and ISRE sequence specific binding regions. Their consensus nucleotides are labeled in red color, and the consensus region is enclosed by the rectangular box. (**B**) Heatmaps display the normalized ChIP-seq reads representing the binding intensity of STAT1 and RelA. Displayed are 8 kb regions centered on the summits of significant STAT1 and/or RelA binding sites. The heatmap are clustered for the STAT1 and RelA binding signal based on the central 0.5 kb of the heatmap. left) Heatmaps of all significant STAT1 and RelA binding sites (n =  13367). right) Heatmap of all significant STAT1 and RelA binding sites that are within 1 kb of a transcription start site (n =  4545). (**C**) Binding of RelA to the promoters of the indicated ISGs. Sequence reads from anti-RelA ChIP-seq or rabbit-IgG-control were plotted relative to chromosomal position. Genome location of corresponding ISGs is shown beneath the track signaling. RelA shows strong and specific binding on the promoters of indicated ISGs, while the rabbit-IgG, serving as negative control, shows no significant binding. (**D**) In the Huh7 cell-based mutant ISRE luciferase reporter cells, TNF-α did not induce mutant ISRE related luciferase activation as measured at 3 different time points (n =  3 independent experiments with 2–3 replicates each). Data presented as mean SD (*P <  0.05; **P <  0.01; ***P <  *0.001;* ns, not significant).

Upon TNF-α stimulation and signaling activation, the transcription factor complex, NF-κ B, can bind to a sequence specific motif [5′ -GGGAA/CTTTCC-3′ ] to promote target gene transcription^13^,^20^,^21^,^22^. The puzzling role of NF-κ B in the transactivation of ISRE led us to perform an *in silico* analysis comparing the ISRE motif and the NF-κ B DNA binding site. Surprisingly, we identified a partial consensus sequence region in common within these two motifs (Fig. 7A). We thus hypothesized that NF-κ B might bind to this consensus sequence within the ISRE motif to drive transcription of corresponding ISGs. To test this hypothesis, we retrieved genome wide RelA and STAT1 (positive control) ChIP-seq data from the ENCODE ChIP-seq Experiment Matrix database. ChIP-seq datasets were processed and analyzed. Confirming our hypothesis, we found that RelA showed a similar genome-wide binding pattern with STAT1. For a large cohort of genes, RelA overlapped with STAT1 in their gene binding site (Fig. 7B, left). To be more specifically, we further analyzed the RelA and STAT1 binding sites that were within 1 kb of a transcription start site. This region is frequently located at the site of the promoter. Consistently, RelA still overlaps with STAT1 in the specific binding sites near gene transcription start sites. Since most genes bound and regulated by STAT1 are ISGs, this indicates that RelA also possesses the ability to bind and regulate a large cohort of ISGs. Then we analyzed RelA binding on a list of well-established antiviral ISGs. Convincingly, RelA shows strong and specific binding on the promoters of indicated ISGs, while the rabbit-IgG (negative control) shows no significant binding (Fig. 7C). To further confirm that NF-κ B binds to the consensus sequence within the ISRE motif to drive corresponding ISG transcription, we mutated the consensus nucleotide sequence within the ISRE motif based on the lentiviral transcriptional reporter vector expressing the firefly luciferase gene driven by multiple ISREs. In theory, RelA will not be able to bind to this mutant ISRE sequence (Supplementary Figure 6). Huh7 cells were transduced with this vector to create a stable reporter cell line. As expected, TNF-α failed to activate this mutated ISRE (Fig. 7D). Hence, NF-κ B can directly bind to the ISRE motif and activate its transcriptional activity.

### TNF-α cooperates with IFN-α in ISG induction and antiviral action

**Figure 8:**
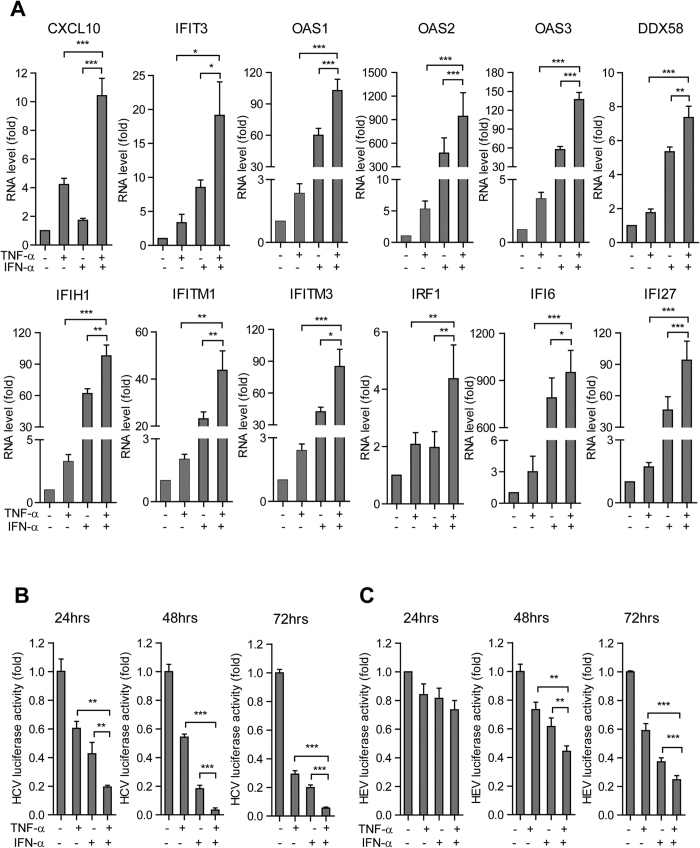
TNF-α cooperates with IFN-α in ISG induction and antiviral action. (**A**) In the Huh7 cells, the combination of TNF-α and IFN-α induced a strong additive ISG expression compared with treatment of either TNF-α or IFN-α alone as measured by qRT-PCR (n =  6). (**B**) In the Huh7 cell-based subgenomic HCV replicon model, the combination of TNF-α and IFN-α induced a strong additive anti-HCV effect compared with treatment of either TNF-α or IFN-α alone as measured at 3 different time (n =  3 independent experiments with 2–3 replicates each). (**C**) Same as (**B**) for the Huh7 cell-based subgenomic HEV replicon model.

Because of the distinct signaling cascades that finally converge the transcription of antiviral ISGs by TNF-α and interferons, we further investigated the combinatory effects of TNF-α with IFN-α on ISG induction and antiviral action. Thus, we quantified the expression levels of a list of well-known antiviral ISGs in the Huh7 cell line with treatment of TNF-α , IFN-α or a combination thereof. Both TNF-α and IFN-α can induce significant up-regulation of tested ISGs, and their combination resulted in a strong additive induction of ISGs (Fig. 8A).

Consistent with a previous publication^23^, our results of ISG antiviral assay (Supplementary Figure 7) again highlight the important antiviral role of ISGs. Thus, the cooperation in ISG induction prompted us to test whether an additive antiviral effect can be achieved with the combination of TNF-α and IFN-α . Hence, we employed the Huh7 cell line based HCV or HEV replicon luciferase reporter as the cell models for the test. As shown in Fig. 8B,C, the combination of TNF-α and IFN-α resulted in additive antiviral effects in both HCV and HEV replicon models. Thus, TNF-α cooperates with IFN-α in ISG induction, explaining their additive antiviral effects against HCV and HEV as we observed.

## Discussion

TNF-α is a cytokine within the TNF superfamily, which acts as a central mediator of inflammation and immune regulations. Although TNF-α was first noted for its role in the killing of tumor cells^24^, it has pleiotropic functions that include the inflammatory response and host resistance to pathogens. Indeed, numerous studies have demonstrated the importance of TNF-α in protection against pathogens, including *Mycobacterium tuberculosis*, *Cryptococcus neoformans*, vesicular stomatitis virus, encephalomyocarditis virus, herpes simplex virus, influenza virus and hepatitis B virus^25^,^26^,^27^,^28^,^29^. Disordered TNF-α regulation may have a significant negative role in inflammation and pathogenesis. Based on this, TNF-α antagonists have been proven to be highly effective in the treatment of certain inflammatory diseases, such as rheumatoid arthritis^30^, psoriatic arthritis^31^, juvenile rheumatoid arthritis^32^, and Crohn’s disease^33^. Several TNF-α inhibitors have been approved for the treatment of these inflammatory illnesses by the US Food and Drug Administration (FDA). Contradictory, many studies have demonstrated an increased risk of opportunistic infections and difficulty in clearing infections once they develop in patients treated with TNF-α inhibitors, such as HBV or HCV infection^34^,^35^,^36^. Our experimental results showing that clinically used anti-TNF-α inhibitors can totally abrogate the antiviral activity of TNF-α appear to support those clinical observations and highlight the primary role of TNF-α in host defense against infections.

As a first line defense, TNF-α and type I interferons are induced by microbial stimuli and mediate innate immune responses. Despite the fact that cells at sites of infection are continuously exposed to both cytokines, the interactions between TNF-α and interferons remain under investigated^37^. Although previous studies have reported that TNF-α interacts with antiviral interferon signaling and regulates ISG expression in the setting of different virus infections^8^,^9^,^10^, the molecular mechanisms behind these interactions have not been delineated. In this study, we demonstrated that the activation of NF-κ B signaling by TNF-α was able to directly transactivate the ISRE motif, resulting in the induction of antiviral ISGs. This whole process is independent of IFN production and the canonical JAK-STAT cascade, but relies on TNF-α induced NF-κ B activity. NF-κ B is a homo- or heterodimeric complex formed by the Rel-like domain-containing proteins: RelA (P65), RelB, c-Rel, P50 and P52 and the heterodimeric RelA (P65)-P50 complex appear to be the most abundant one. The dimers bind to the sequence specific NF-κ B response element in the promoter region of their target genes to regulate transcription. To our surprise, *in silico* analysis discovered a consensus nucleotide sequence shared by the ISRE motif and NF-κ B DNA binding site. ChIP-seq data analysis reveals RelA (P65) can directly bind to the promoter region of a large cohort of ISGs. Our loss-of-function and mutagenesis assay further confirmed that NF-κ B could directly drive ISRE-controlled gene transcription. Since NF-κ B is also the key downstream effector of most Toll-like receptors (TLR), this novel mechanism may also partially explain the antiviral activities of TLR agonists in clinic, such as the TLR7 agonists, which are being therapeutically targeted and explored for HCV treatment in clinic trial^38^.

More excitingly, TNF-α not only activates antiviral ISGs transcription, but also cooperates with IFN-α , explaining the additive antiviral outcome of their combination. This highlights the important facts that different cytokines orchestrate innate immune responses by activating signaling cascades to protect against infection efficiently.

In conclusion, we revealed a novel antiviral mechanism of TNF-α . TNF-α ,via the activation of NF-κ B cascade, can drive the transcription of antiviral ISGs through direct binding of ISREs. This antiviral mechanism may provide clues for tackling the high rise of infections caused by TNF-α inhibitor treatment in patients. More interestingly, TNF-α also acts cooperatively with IFN-α in antiviral ISGs induction to exert additive antiviral effects. These findings not only provide new clues for understanding virus-host interactions but also assign a novel function of the canonical NF-κ B pathway.

## Materials and Methods

The HCV subgenomic replicon comprised Huh7 cells containing a subgenomic HCV bicistronic replicon (1389/NS3-3 V/LucUbiNeo-ET) linked to the firefly luciferase reporter gene were maintained with 250 μ g/ml G418 (Sigma, Zwijndrecht, the Netherlands). The HEV subgenomic model was based on Huh7 cells containing the subgenomic HEV sequence (Kernow-C1 p6/luc) coupled to a Gaussia luciferase reporter gene. Lentiviral pLKO knockdown vectors (Sigma–Aldrich) targeting IRF1, TNFR1, RelA were obtained from the Erasmus Biomics Center and produced in HEK293T cells as previously described^39^. The use of serum samples from IBD patients was approved by the Medical Ethical Committee of the Erasmus Medical Center (Medisch Ethische Toetsings Commissie Erasmus MC), and the informed consent was obtained from all subjects. All methods were carried out in accordance with the approved guidelines. For more details, see Supplementary Information.

## Additional Information

**How to cite this article**: Wang, W. *et al.* Convergent Transcription of Interferon-stimulated Genes by TNF-α and IFN-α Augments Antiviral Activity against HCV and HEV. *Sci. Rep.* **6**, 25482; doi: 10.1038/srep25482 (2016).

## Supplementary Material

Supplementary Information
