# Supplementary material for: Convergent Transcription of Interferon-stimulated Genes by TNF-α and IFN-α Augments Antiviral Activity against HCV and HEV
Source: Sci Rep. 2016 May 6;6:25482. doi: 10.1038/srep25482 (PMC4858707; doi:10.1038/srep25482)
Supplement: Supplementary Information [file srep25482-s1.doc]

**Supplementary Information**

**Convergent Transcription of Interferon-stimulated Genes by TNF-α and IFN-α Augments Antiviral Activity against HCV and HEV**

Wenshi Wang, Lei Xu, Johannes H Brandsma, Yijin Wang, [Mohamad S. Hakim](javascript:void(0)), Xinying Zhou, Yuebang Yin, Gwenny M. Fuhler, Luc J. W. van der Laan, C. Janneke van der Woude, Dave Sprengers, Herold J. Metselaar, Ron Smits, Raymond A. Poot, Maikel P. Peppelenbosch and Qiuwei Pan

**Supplementary Materials and Methods**

**Reagents**

Recombinant human TNF-α (Peprotech, USA) and human IFN-α (Thermo Scientific, the Netherlands) was dissolved in PBS. Stocks of JAK inhibitor 1 (Santa Cruz Biotech, CA) and Bayer-18 (Synkinase, China) were dissolved in DMSO with a final concentration of 5 mg/mL. Antibodies phospho-STAT1 (Tyr701) (58D6, #9167), STAT1 (#9172), RelA (P65) (C22B4, #4764), IRF1 (D5E4), IRF7 (D2A1J), Anti-rabbit IgG(H+L),F(ab') 2 Fragment (Alexa Fluor 488 conjugate) and Anti-mouse IgG (H+L), F(ab')2 Fragment (Alexa Fluor® 488 Conjugate) were purchased from Cell Signaling Technology. IRF9 antibody was obtained from LSBio (Life Span BioSciences, Inc). β-actin, STAT2 (sc-476), phospho-STAT2 (Tyr690) were purchased from Santa Cruz Biotechnology; anti-rabbit or anti-mouse IRDye-conjugated antibodies were used as secondary antibodies for western blotting (Stressgen, Victoria, BC, Canada).

**Cell models**

The HCV subgenomic replicon comprised Huh7 cells containing a subgenomic HCV bicistronic replicon (1389/NS3-3V/LucUbiNeo-ET) linked to the firefly luciferase reporter gene were maintained with 250 μg/ml G418 (Sigma, Zwijndrecht, the Netherlands). The HEV subgenomic model was based on Huh7 cells containing the subgenomic HEV sequence (Kernow-C1 p6/luc) coupled to a Gaussia luciferase reporter gene. Luciferase normalization cells (LV-PGK-Luc) were generated by transducing Huh7 cells with a lentiviral vector expressing the firefly luciferase gene under control of the human phosphoglycerate kinase (PGK) promoter. ISRE, NF-κB, AP-1 luciferase reporter cells were generated by transducing Huh7 cells with lentiviral vectors expressing the firefly luciferase gene under the control of the promoters containing the ISRE, NF-κB, AP-1 motifs, respectively (System Biosciences).

**Gene knockdown or over-expression by lentiviral vectors**

Lentiviral pLKO knockdown vectors (Sigma–Aldrich) targeting IRF1, IRF7, STAT1, TNFR1, RelA (P65) were obtained from the Erasmus Biomics Center and produced in HEK293T cells. After a pilot study, the shRNA vectors exerting optimal gene knockdown were selected. These shRNA sequences are listed in Supplementary Table 1. Stable gene knockdown cells were generated after lentiviral vector transduction and puromycin (2 μg/ml; Sigma) selection. IRF1, IFI6 and DDX58 lentiviral overexpression vectors were a kind gift from Prof. Charles M. Rice, the Rockefeller University [^1^](#_ENREF_1). Meanwhile, two control vectors expressing reporter genes *Photinus pyralis* luciferase (Fluc) or Green fluorescent protein (GFP) were also used.

**Measurement of luciferase activity**

For *Gaussia* luciferase analysis, the activity of secreted luciferase in the cell culture medium was measured by BioLux® *Gaussia* Luciferase Flex Assay Kit (New England Biolabs) according to the manufacturer’s instructions. For firefly luciferase, luciferin potassium salt (100 mM; Sigma) was added to cells and incubated for 10 min at 37 °C The luciferase activity was quantified with a LumiStar Optima luminescence counter (BMG Lab Tech, Offenburg, Germany).

**Quantitative real-time polymerase chain reaction**

RNA was isolated with a Machery-NucleoSpin RNA II kit (Bioke, Leiden, The Netherlands) and quantified using a Nanodrop ND-1000 (Wilmington, DE, USA). cDNA was synthesized from total RNA using a cDNA Synthesis Kit (TAKARA BIO INC). The cDNA of all detected genes was amplified for 50 cycles and quantified with a SYBRGreen-based real-time PCR (Applied Biosystems) according to the manufacturer’s instructions. GAPDH and RP2 were considered as reference genes to normalize gene expression. All the primer sequences are included in Supplemental Table 2.

**Western Blot Assay**

Cultured cells were lysed in Laemmli sample buffer containing 0.1 M DTT and heated 5 mins at 95 °C, followed by loading onto a 10% sodium dodecyl sulfate polyacrylamide gel and separation by electrophoresis. After 90 mins running at 120 V, proteins were electrophoretically transferred onto a polyvinylidene difluoride membrane (Invitrogen) for 1.5 hrs with an electric current of 250 mA. Subsequently, the membrane was blocked with a mixture of 2.5 ml blocking buffer (Odyssey) and 2.5 ml phosphate-buffered saline containing 0.05% Tween 20. It was followed by overnight incubation with primary antibodies (1:1000) at 4 °C. The membrane was washed 3 times followed by incubation for 1h with IRDye-conjugated secondary antibody (1:5000). After washing 3 times, protein bands were detected with the Odyssey 3.0 Infrared Imaging System.

**Enzyme-linked immunosorbent assay (ELISA)**

Serum samples were collected and stored at - 80 °C. TNF-α level was measured by an ELISA kit (eBioscience, USA) according to manufacturer’s instructions. The absorbance was measured at 450 nm in an automatic microplate reader. Results were calculated based on a standard curve.

**Confocal laser electroscope assay**

Huh7 cells were seeded on glass coverslips. After 12 hrs, cells were washed with PBS, fixed in 4% PBS-buffered formalin for 10 mins and blocked with tween-milk-glycine medium (PBS, 0.05% tween, 5g/L skim milk and 1.5g/L glycine). Samples were incubated with primary antibodies overnight at 4 °C. Subsequently, samples were incubated with 1:1000 dilutions of the anti-mouse IgG (H+L), F(ab')2 Fragment (Alexa Fluor® 488 Conjugate) or anti-rabbit IgG(H+L), F(ab') 2 Fragment (Alexa Fluor 488 conjugate) secondary antibodies. Nuclei were stained with DAPI (4,6-diamidino-2-phenylindole; Invitrogen). Images were detected using confocal electroscope.

**ChIP-seq data analysis**

ChIP-seq datasets for STAT1 in Gm12878 cells and RelA in the TNFα stimulated Gm12878 cells were retrieved from the ENCODE database. ChIP-seq datasets were processed and mapped to hg38 reference genome as described [^2^](#_ENREF_2). ChIP-seq datasets with multiple replicates were merged. MACS 1.4.2 was used for peak calling and for the generation of binding profiles [^3^](#_ENREF_3). MACS was run with -p 1e-10, using the mock control of TNF-α stimulated cell as control dataset for both the STAT1 and RelA ChIP-seq. Heatmaps were generated based on a unified peak list. If the centers of two binding regions reported by MACS were 100 bp or less apart, they were unified to a single binding region. Heatmaps were normalized for each individual factor by calculating the RPM based on the sum of all reads displayed by the heatmap. RPM was log2 transformed and manhattan clustering was performed. The heatmap images were generated in R. The sequencing profiles were generated in the IGV browser [^4^](#_ENREF_4).

**Construction of mutant ISRE reporter cell line**

Based on the sequence specific ISRE motif, a mutant version of ISRE was designed and synthesized (Forward: 5’- *aattc*AGTTTCGTCAAGTCTTTCAGTTTCGTCAAGTCTTTCAGTTTCGTCAAGTCTTTCAGTTTCGTCAAGTCTTT*a* -3’; Reverse: 5’- *ctagt*AAAGACTTGACGAAACTGAAAGACTTGACGAAACTGAAAGACTTGACGAAACTGAAAGACTTGACGAAACT*g* -3’), which shows no consensus sequence with the NF-κB motif. EcoRI and SpeI sites are included (shown in italics) to facilitate directional cloning into the pGreenFire Lenti-Reporter vector (System Biosciences). The recombinant plasmid was verified by restriction enzyme digestion and DNA sequencing. Stable mutant ISRE reporter cells were generated after lentiviral vector transduction and puromycin (2 μg/ml; Sigma) selection.

**Statistical analysis**

All results were presented as mean ± SD. Comparisons between groups were performed with Mann-Whitney test. Differences were considered significant at a P value less than 0.05.

**Ethics Statement**

The use of serum samples from IBD patients was approved by the Medical Ethical Committee of the Erasmus Medical Center (Medisch Ethische Toetsings Commissie Erasmus MC). The volunteers or patients agreed to participate by written informed consent.

**Supplementary Figure and Figure Legends**

**
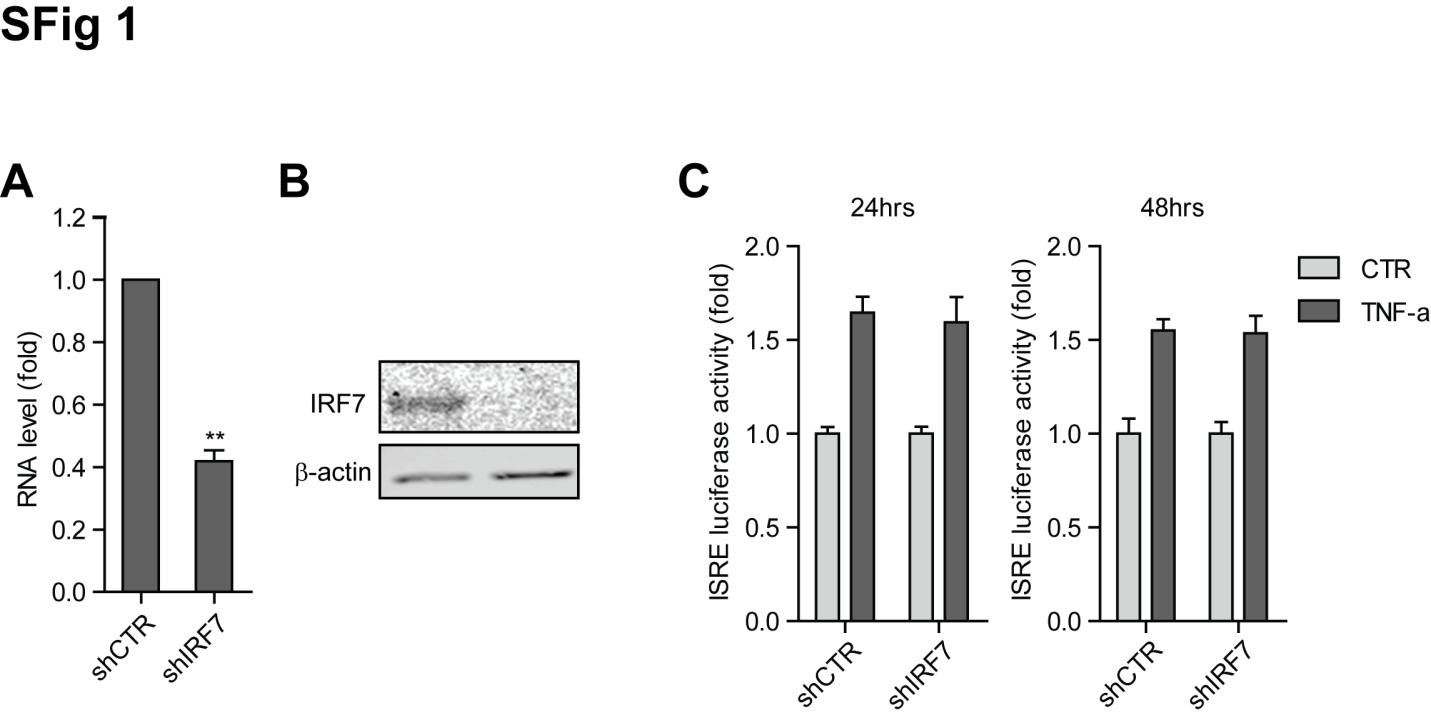
**

**Figure S1. TNF-α induced ISRE activation is independent of IRF7.**

(A) qRT-PCR analysis of successful IRF7 knockdown by lentiviral shRNA vectors in the Huh7 based ISRE luciferase reporter cells.

(B) Western blot analysis confirmed the successful knockdown of IRF7 by lentiviral shRNA vectors in the Huh7 based ISRE luciferase reporter cells.

(C) IRF7 knockdown had no significant influence on TNF-α induced ISRE-related luciferase activation as measured at 2 different time points (n = 3 independent experiments with 2 – 3 replicates each).


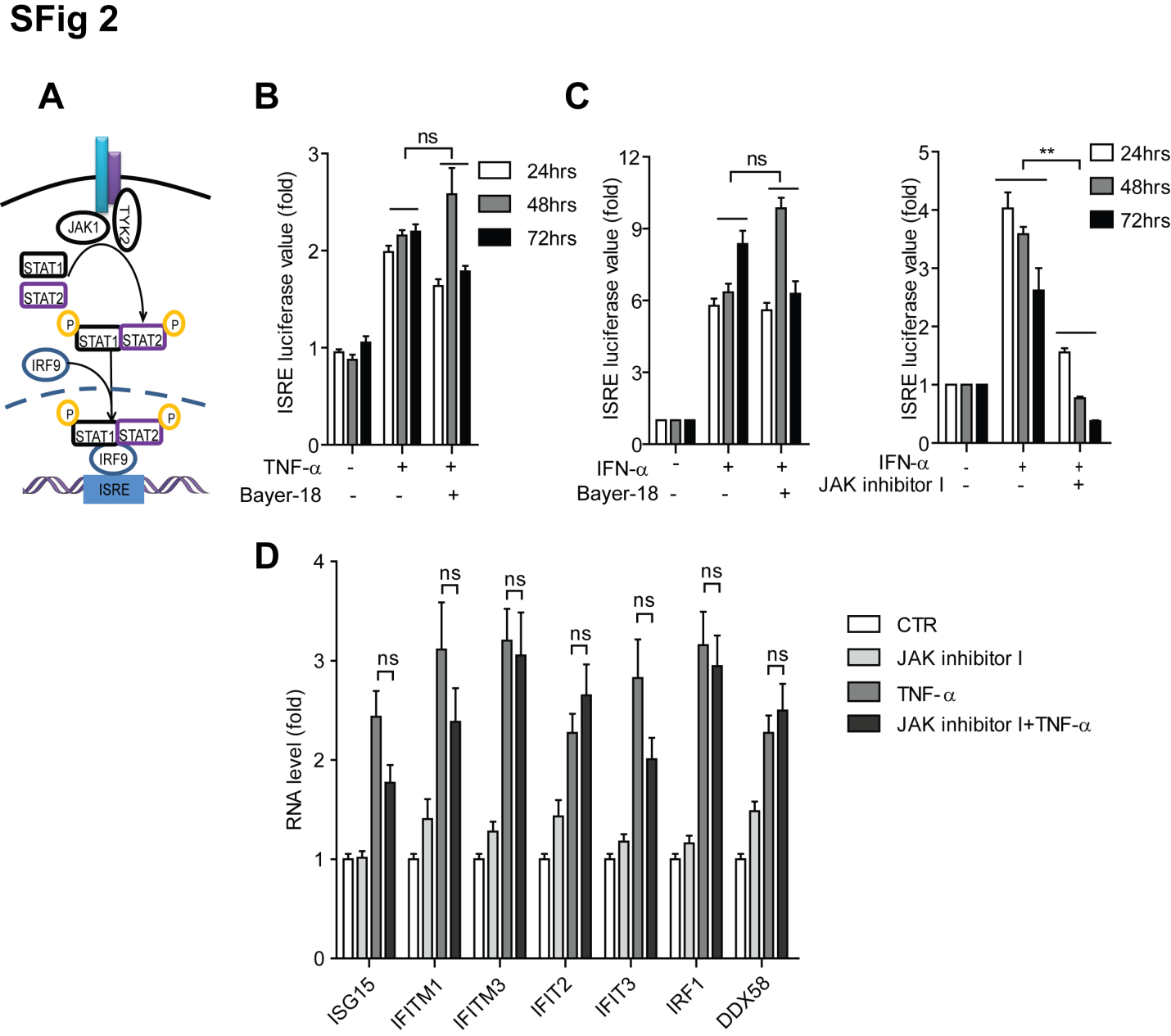


**Figure S2. TNF-α induced ISRE activation is independent of interferon and the JAK-STAT signaling**

(A) Illustration of key elements in IFN-α induced JAK-STAT signaling pathway.

(B) The selective TYK2 inhibitor, Bayer-18, did not abrogate TNF-α induced ISRE-related luciferase activation (n = 3 independent experiments with 2 – 3 replicates each).

(C) In Huh7 based ISRE luciferase cells, TYK2 selective inhibitor, Bayer-18, did not exert significant effect on IFN-α (1000 IU/ml) induced ISRE-luciferase activity (left), while JAK inhibitor I (10 μM) abrogated IFN-α (1000 IU/ml) induced ISRE luciferase activity as measured at 24, 48 and 72 hrs (n = 3 independent experiments with 2 – 3 replicates each).

(D) JAK inhibitor I exerts no significant influence on TNF-α induced ISG expression as measured by qRT-PCR. (n = 4).


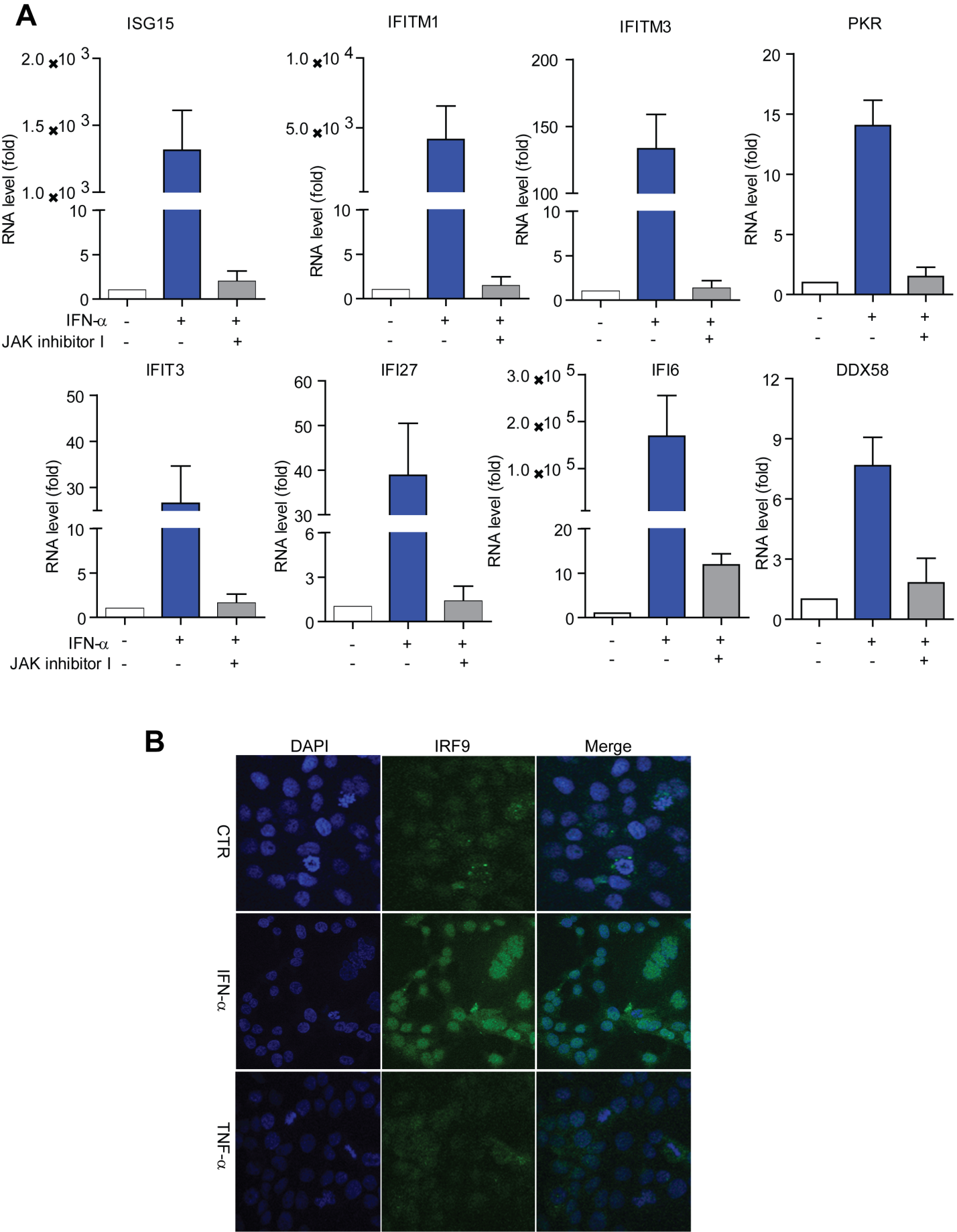


**Figure S3. IFN-α induced ISRE activation depends on the JAK-STAT signaling**

(A) In Huh7 cells, JAK inhibitor I (10 μM) abrogated IFN-α (1000 IU/ml) induced ISG expression as measured by qRT-PCR.

(B) Confocal microscopy analysis of IRF9 localization in Huh7 cells treated with IFN-α or TNF-α. IRF9 was induced and translocated to the nucleus upon IFN-α, but not TNF-α treatment. IRF9 antibody (green). Nuclei were visualized by DAPI (blue).

**
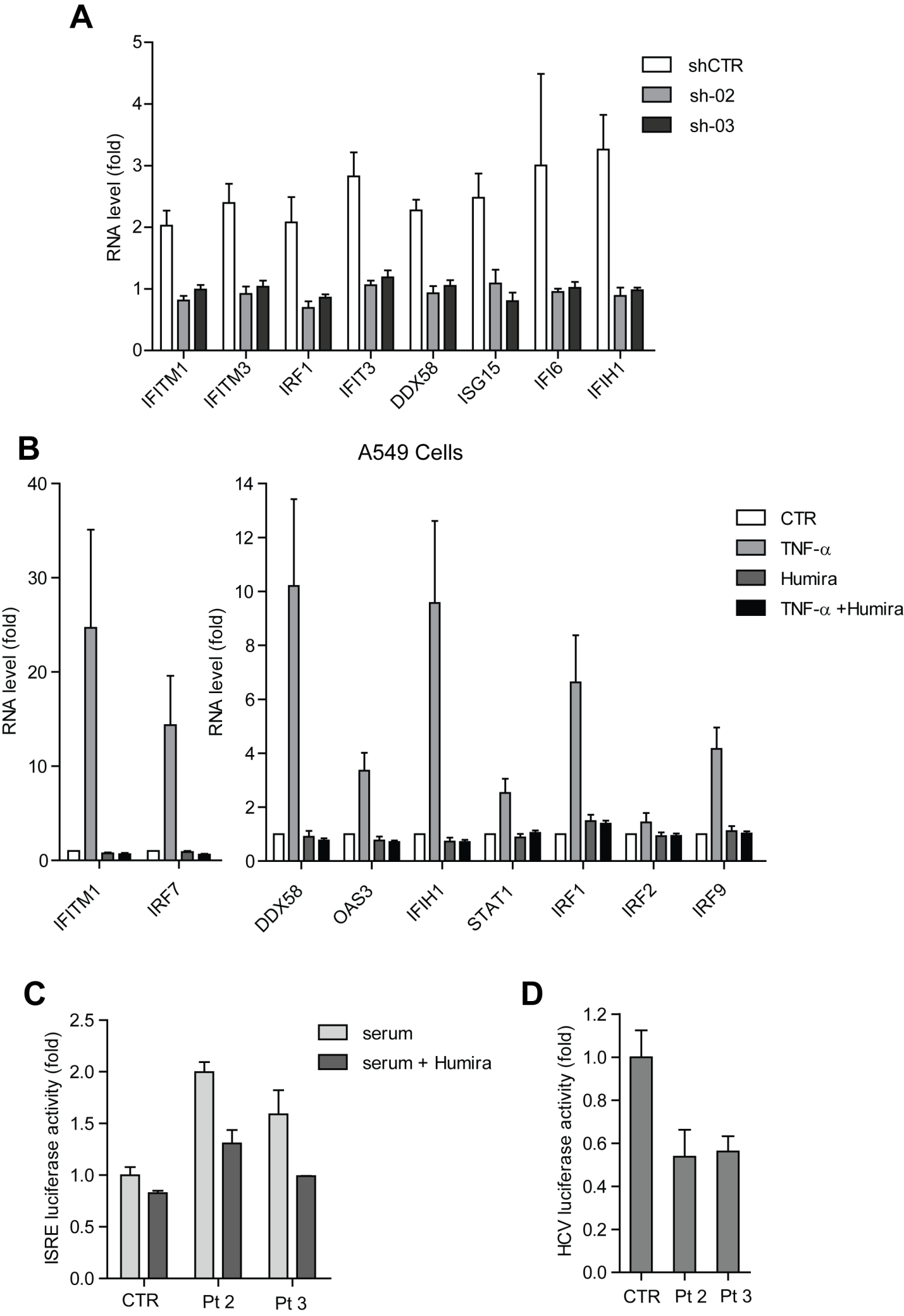
**

**Figure S4. TNF-α activates ISRE via TNF receptor**

(A) TNFR1 knockdown blocked TNF-α induced ISG expression as measured by qRT-PCR (n = 4).

(B) In A549 cells, TNF-α inhibitor, Humira, abrogated TNF-α induced ISG expression as measured by qRT-PCR (n = 4).

(C) Humira decreased serum samples (with higher TNF-α levels) induced ISRE-related luciferase activity.

(D) Serum samples with higher TNF-α levels inhibited HCV-related luciferase activity compared with control serum.

**
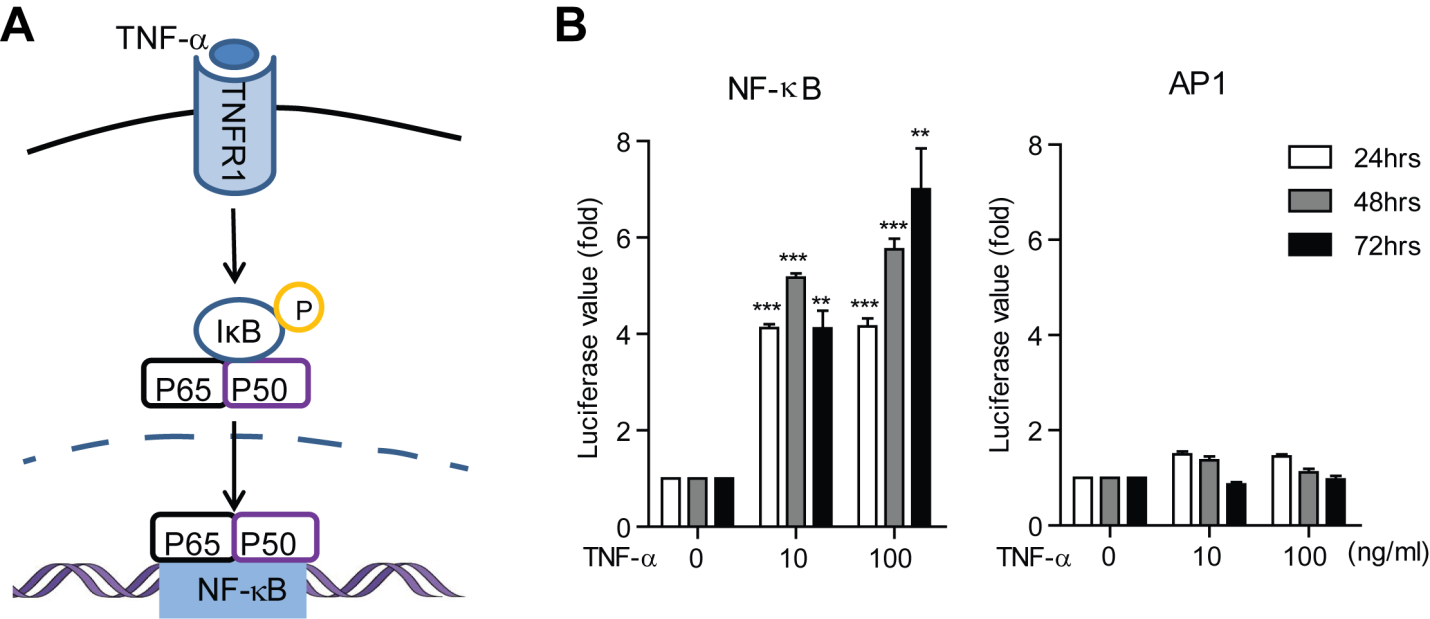
**

**Figure S5. TNF-α efficiently activate NF-**κ**B signaling pathway**

(A) Illustration of key elements in TNF-α induced NF-κB signaling pathway.

(B) In the Huh7 cell-based NF-κB or AP1 luciferase reporter cells, TNF-α dose-dependently induced activation of NF-κB-related luciferase activity, while no significant effect on AP1-related luciferase activity as measured at 3 different time points (n = 3 independent experiments with 2 – 3 replicates each).


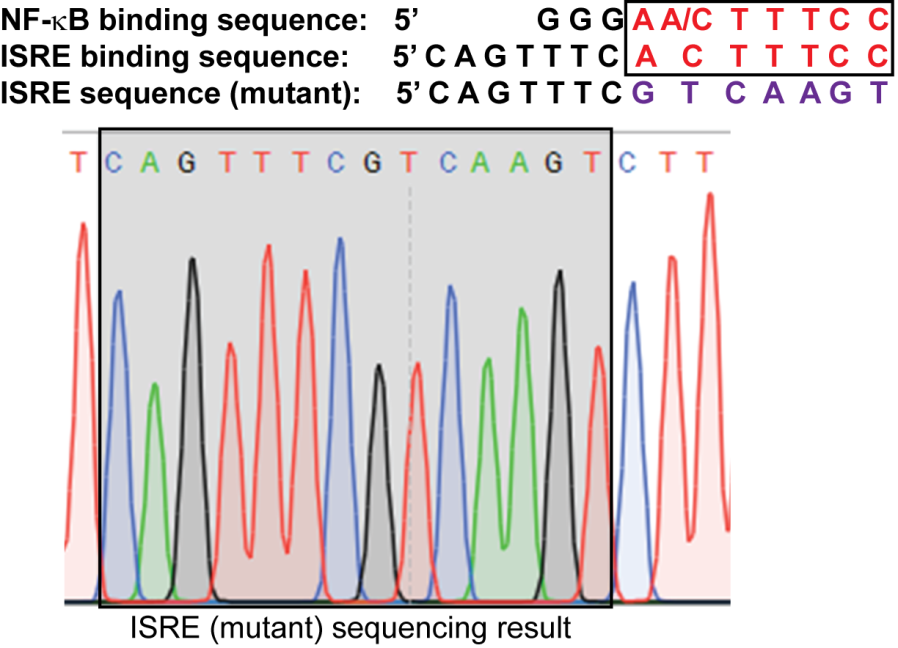


**Figure S6. The nucleotide sequence of NF-**κ**B, ISRE and the ISRE mutant binding regions.** Their consensus nucleotides are labeled in red color, and the consensus region is marked in a rectangular box. The mutated nucleotides are shown in purple color. The ISRE (mutant) sequencing result is shown in the illustration below.


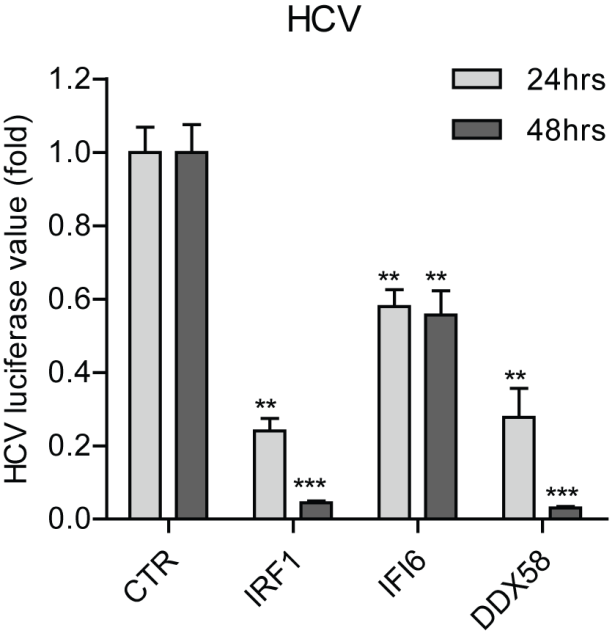


**Fig. S7. ISG, e.g. IRF1, IFI6 or DDX58 exerts strong antiviral effect against HCV.**

Huh7 cell based HCV replicon luciferase reporter was transduced with integrating lentiviral vectors to overexpress ISG, e.g. IRF1, IFI6 or DDX58, showing strong antiviral potency against HCV.

**Table S1. shRNA sequences**

| Name | Oligo Sequences |
| --- | --- |
| shIRF1 | CCGGGCGTGTCTTCACAGATCTGAACTCGAGTTCAGATCTGTGAAGACACGCTTTTT |
| shTNFR1-01 | CCGGCATTGGTTTAATGTATCGCTACTCGAGTAGCGATACATTAAACCAATGTTTTTG |
| shTNFR1-02 | CCGGGCTTGAAGGAACTACTACTAACTCGAGTTAGTAGTAGTTCCTTCAAGCTTTTTG |
| shTNFR1-03 | CCGGGTGCCACAAAGGAACCTACTTCTCGAGAAGTAGGTTCCTTTGTGGCACTTTTTG |
| shRelA | CCGGCCTGAGGCTATAACTCGCCTACTCGAGTAGGCGAGTTATAGCCTCAGGTTTTT |
| shIRF7 | CCGGCCCGAGCTGCACGTTCCTATACTCGAGTATAGGAACGTGCAGCTCGGGTTTTT |
| ShSTAT1 | CCGGGAACAGAAATACACCTACGAACTCGAGTTCGTAGGTGTATTTCTGTTCTTTTT |

**Table S2. Primer sequences**

| Gene | Sequences 5’ to 3’ |
| --- | --- |
| DDX58-F | CACCTCAGTTGCTGATGAAGGC |
| DDX58-R | GTCAGAAGGAAGCACTTGCTACC |
| ISG15-F | CTCTGAGCATCCTGGTGAGGAA |
| ISG15-R | AAGGTCAGCCAGAACAGGTCGT |
| STAT2-F | CAGGTCACAGAGTTGCTACAGC |
| STAT2-R | CGGTGAACTTGCTGCCAGTCTT |
| IFITM3-F | CTGGGCTTCATAGCATTCGCCT |
| IFITM3-R | AGATGTTCAGGCACTTGGCGGT |
| STAT1-F | ATGGCAGTCTGGCGGCTGAATT |
| STAT1-R | CCAAACCAGGCTGGCACAATTG |
| IFI27-F | CGTCCTCCATAGCAGCCAAGAT |
| IFI27-R | ACCCAATGGAGCCCAGGATGAA |
| PKR-F | GAAGTGGACCTCTACGCTTTGG |
| PKR-R | TGATGCCATCCCGTAGGTCTGT |
| IFIH1-F | GCTGAAGTAGGAGTCAAAGCCC |
| IFIH1-R | CCACTGTGGTAGCGATAAGCAG |
| IFIT2-F | GGAGCAGATTCTGAGGCTTTGC |
| IFIT2-R | GGATGAGGCTTCCAGACTCCAA |
| IFIT3-F | CCTGGAATGCTTACGGCAAGCT |
| IFIT3-R | GAGCATCTGAGAGTCTGCCCAA |
| IRF1-F | GAGGAGGTGAAAGACCAGAGCA |
| IRF1-R | TAGCATCTCGGCTGGACTTCGA |
| IRF9-F | CCACCGAAGTTCCAGGTAACAC |
| IRF9-R | AGTCTGCTCCAGCAAGTATCGG |
| IFIT1-F | GCCTTGCTGAAGTGTGGAGGAA |
| IFIT1-R | ATCCAGGCGATAGGCAGAGATC |
| IFIT2-F | GGAGCAGATTCTGAGGCTTTGC |
| IFIT2-R | GGATGAGGCTTCCAGACTCCAA |
| CXCL10-F | GGTGAGAAGAGATGTCTGAATCC |
| CXCL10-R | GTCCATCCTTGGAAGCACTGCA |
| MX1-F | GGCTGTTTACCAGACTCCGACA |
| MX1-R | CACAAAGCCTGGCAGCTCTCTA |
| IRF2-F | TAGAGGTGACCACTGAGAGCGA |
| IRF2-R | CTCTTCATCGCTGGGCACACTA |
| IRF7-F | CCACGCTATACCATCTACCTGG |
| IRF7-R | GCTGCTATCCAGGGAAGACACA |
| IRF9-F | CCACCGAAGTTCCAGGTAACAC |
| IRF9-R | AGTCTGCTCCAGCAAGTATCGG |
| IFNα-F | 5'-GACTCCATCTTGGCTGTGA-3' |
| IFNα-R | 5'-TGATTTCTGCTCTGACAACCT-3' |
| IFNβ1-F | CTTGGATTCCTACAAAGAAGCAGC |
| IFNβ1-R | TCCTCCTTCTGGAACTGCTGCA |
| TNFR1-F | CCGCTTCAGAAAACCACCTCAG |
| TNFR1-R | ATGCCGGTACTGGTTCTTCCTG |

**Referneces**

1. Schoggins JW*, et al.* A diverse range of gene products are effectors of the type I interferon antiviral response. *Nature* **472**, 481-485 (2011).

2. Engelen E*, et al.* Proteins that bind regulatory regions identified by histone modification chromatin immunoprecipitations and mass spectrometry. *Nat Commun* **6**, 7155 (2015).

3. Zhang Y*, et al.* Model-based analysis of ChIP-Seq (MACS). *Genome Biol* **9**, R137 (2008).

4. Robinson JT*, et al.* Integrative genomics viewer. *Nat Biotechnol* **29**, 24-26 (2011).
